# Editorial: Animal models, gut microbiota and brain diseases

**DOI:** 10.3389/fmicb.2025.1653756

**Published:** 2025-07-24

**Authors:** Yu Wang, Jin Song, Chang Liu, Niraj Kumar Jha, Kavindra Kumar Kesari

**Affiliations:** ^1^Institute of Acupuncture and Moxibustion, China Academy of Chinese Medical Sciences, Beijing, China; ^2^Beijing Hospital of Traditional Chinese Medicine, Capital Medical University, Beijing, China; ^3^Beijing Institute of Chinese Medicine, Beijing, China; ^4^State Key Laboratory of Microbial Technology, Shandong University, Qingdao, China; ^5^Department of Biotechnology and Bioengineering, School of Biosciences and Technology, Galgotias University, Greater Noida, India; ^6^Aalto University, Otakaari, Finland; ^7^University Center for Research and Development, Chandigarh University, Mohali, Punjab, India

**Keywords:** gut microbiota, brain diseases, microbiota-gut-brain axis, interventions, animals

## Introduction

Brain diseases, including neurological and psychiatric disorders such as Alzheimer's, Parkinson's, epilepsy, depression, anxiety, autism, insomnia, etc., significantly impact human health. These conditions are mainly characterized by abnormal thinking patterns, cognition, emotional states, and behavior (Gao et al., 2023). Research primarily relies on animal models, but the pathophysiology remains incompletely understood. Determining whether findings are causal, related, or irrelevant is critical for advancing effective treatments.

There is a need for updated insights from preclinical animal studies. Since 2011, research has shown that germ-free mice exhibit reduced anxiety-like behavior and altered neurochemicals, sparking interest in the gut microbiota's role in health (Neufeld et al., 2011). The concept of the microbiota-gut-brain axis (MGBA), formalized in 2012, highlights the connection between peripheral systems and the brain via gut microbiota (Cryan and Dinan, 2012). Over the past decade, animal models have been crucial in investigating the gut microbiota's impact on brain diseases and potential therapies. However, applying these findings to human brain disease diagnosis and treatment remains challenging.

The aim of this Research Topic was to compile new studies on animal models, gut microbiota, and brain diseases. It features 23 articles: 15 original research papers, 4 reviews, 2 systematic reviews, and 2 mini reviews.

## Neurodegenerative disorders

### Dementia and cognitive impairment

Researchers have long debated the link between gut microflora and dementia. Fu J. et al. conducted a Mendelian randomization (MR) study and identified 21 gut microbiota taxa linked to dementia subtypes. *Desulfovibrionaceae* was associated with a higher risk of Alzheimer's, while *Butyricimonas* was linked to a lower risk of Parkinson's dementia. A bidirectional relationship was observed between Ruminococcus gnavus and Lewy body dementia. Sensitivity analyses confirmed these results, though the study's limitations include relaxed SNP thresholds and a lack of ethnic diversity, as most data came from European populations. These findings indicate gut microbiota could be key in diagnosing and treating dementia. Yang et al. examined the impact of inflammatory cytokines and gut microbiota on the risk of vascular dementia (VaD) and their causal links. Their MR study found causal links between specific gut microbiota, like *Negativicutes* (beneficial) and *Melainabacteria* (detrimental), and inflammatory cytokines, such as IL-18 and MIF (risk-increasing) and IL-4 (beneficial), with different types of vascular dementia. Multivariable analyses confirmed the microbiome effects were independent of factors like body mass index, while mediation MR analyses dismissed cytokines as intermediaries between the gut and VaD. These results highlight new microbial and immune targets for VaD treatment and improve our understanding of the gut-brain connection in cerebrovascular cognitive decline.

The above two MR studies highlight the significant role of intestinal microbiota in the pathogenesis of dementia. This raises the question of what therapeutic interventions are available for dementia and cognitive impairment within this framework. Dong et al. demonstrated that fecal microbiota transplantation (FMT) improved cognitive function in rats with traumatic brain injury (TBI) from gas explosions by restoring gut microbial balance (e.g., *Clostridium_T, Allobaculum*) and strengthening the gut-brain barrier. FMT reduced neuroinflammation, increased tight-junction proteins like Claudin-1, Occludin, and ZO-1, and affected Treg-related factors. Multi-omics analyses revealed fatty acid biosynthesis activation as a key mechanism. These results highlight FMT's potential as a TBI treatment by modulating the MGB axis. Similarly, Qi et al. found that Total Alkaloids of Rhizoma Corydalis (TAC) ameliorated cognitive function by balancing gut microbiota, enhancing intestinal barrier integrity, and reducing neuroinflammation. TAC decreases *Lachnoclostridium*, increases *Bacteroides*, upregulates ZO-1 and occludin, and inhibits hippocampal NLRP3 inflammasome activation and neuronal ferroptosis.

Additionally, three reviews summarize research on gut microbiota and cognitive impairment in dementia. Tang et al. linked intermittent hypoxia from obstructive sleep apnea (OSA) to gut dysbiosis, marked by fewer short-chain fatty acid (SCFA) producers and more *Prevotella* species, as well as to systemic inflammation and cognitive decline. FMT from OSA-model mice into healthy mice replicated these effects, confirming a causal link. They suggest prebiotic, probiotic, or SCFA treatments could help mitigate OSA-related neural damage. Abildinova et al. emphasized the role of microbiota-derived short-chain fatty acids, like butyrate, in regulating insulin sensitivity and cognitive function. Dysbiosis can lead to metabolic endotoxemia, marked by lipopolysaccharide translocation, systemic inflammation, and brain insulin resistance, which may speed up cognitive decline. Potential therapies include multi-strain probiotics, personalized nutrition, and microbiota-derived exosomes. While FMT and probiotics show promise in restoring metabolic-cognitive balance, challenges such as outcome variability and long-term safety remain. Future research should aim to clarify mechanisms and develop personalized microbial therapies. Ba et al. identify the MGBA as a promising target for treating post-stroke cognitive impairment (PSCI), emphasizing acupuncture's role in enhancing intestinal barrier function, gut microbiota, and reducing neuroinflammation. Clinical studies demonstrated that acupuncture at specific points (e.g., GV20, ST36) improves cognitive scores, and research indicates its modulatory effects on metabolites, immune markers, and HPA axis activity. However, the lack of high-quality trials and standardized protocols remains a significant challenge. Future research should focus on integrating multi-omics and refining acupuncture techniques for precise interventions in PSCI.

### Parkinson's disease

Zeng J. et al. investigated transplantation of gut microbiota from individuals with Parkinson's disease (PD) and healthy controls (HC) were into germ-free honeybees. The results indicate that fecal microbiota transplants from PD patients result in motor impairments, reduced expression of tyrosine hydroxylase, and compromised gut barrier integrity in honeybees, paralleling the pathology observed in rotenone-induced PD models. This “humanized bee model” exhibited enrichment of PD-associated genera (e.g., *Dorea, Collinsella*), and altered microbial pathways involved in hydrogen sulfide and methane production, thereby underscoring the significance of the MGBA in PD pathogenesis. Despite certain translational limitations, this innovative invertebrate model offers a rapid and ethical approach to exploring the mechanistic aspects of gut-brain interactions.

As for therapy, emerging evidence indicates that electroacupuncture (EA) may benefit PD, though its mechanisms are not well understood. Hu et al. demonstrated that EA at ST25 improved motor function and reduced neuron loss in a PD rat model induced by rotenone. EA also corrected gut dysbiosis by decreasing harmful bacteria and increasing beneficial ones, lowering inflammation and lipid peroxidation in the brain. These results highlight the GMBA as a potential non-drug treatment pathway for PD.

### Epilepsy

Using ciprofloxacin-induced seizure susceptibility and lithium pilocarpine-induced epilepsy rat models, Zou et al. elucidated that extended administration of ciprofloxacin results in gut microbiota dysbiosis. This dysbiosis, characterized by increased *Akkermansia* and *Bacteroides* abundance, is associated with enhanced seizure susceptibility in rats. Critically, FMT reversed both the microbial dysbiosis and the pro-epileptogenic effects. Furthermore, reduced serum indole-3-propionic acid levels were identified as a potential driver of neuroinflammation. These findings provide compelling evidence supporting the role of the MGBA in the pathogenesis of epilepsy and suggest that gut microbiota modulation represents a therapeutically viable. Although clinical translation requires further validation, this research provides crucial mechanistic insights into the risks associated with antibiotic-induced seizures and potential microbial-targeted intervention.

## Psychiatric disorders

### Depression

Xie et al.'s review highlights the pivotal role of gut microbiota in modulating antidepressant treatment responses, associating baseline microbial profiles (e.g., *Firmicutes/Bacteroidetes* ratio) with clinical outcomes via immune-neural interactions. Although these findings are promising, limitations including small sample sizes constrain generalizability and require further validation. Microbiota-targeted therapies offer potential for advancing personalized approaches to depression management. In this regard, the study by Wang J. et al. illustrates that electroacupuncture at points ST36 and ST25 alleviates depressive-like behaviors in rats subjected to chronic unpredictable mild stress by modulating gut microbiota (e.g., increased *Bacteroidetes* and decreased *Firmicutes*) and neuropeptides (VIP/CGRP). These findings implicate the MGBA in the antidepressant effects of acupuncture, while the precise neural mechanisms require further elucidation.

### Anxiety

Xu et al. conducted a Mendelian randomization study revealing that certain gut microbiota genera affect anxiety disorders. The *Eubacterium nodatum* group and *Ruminococcaceae* UCG011 are protective, while *Lachnospiraceae* UCG010 elevates risk. These effects are linked to neurotransmitter-related metabolites like tyrosine, phenylalanine, glycine, and cortisol. The study highlights the influence of modifiable factors, such as diet, smoking, and physical activity, on microbiota-anxiety interactions, suggesting potential for precision anxiety management through targeted lifestyle interventions.

### Insomnia

Wang X. et al. conducted a bidirectional Mendelian randomization study establishing causal links between specific gut microbiota and sleep traits. The analysis revealed that class *Negativicutes* and order *Selenomonadales* elevate severe insomnia risk, whereas phylum *Lentisphaerae* is causally associated with longer sleep duration, and genus *Senegalimassilia* reduces snoring propensity. Reverse MR analysis demonstrated that sleep patterns reciprocally alter microbiota composition. The study's strengths include multivariable sensitivity analyses and STROBE-MR guideline adherence, though limitations encompass ethnic homogeneity and relaxed SNP thresholds. Collectively, these findings suggest targeting microbiota could help manage sleep disorders. Guo et al.'s review further implicates the MGBA as key in insomnia's pathogenesis, focusing on dysbiosis-related disruptions in various signaling pathways. It identifies acupuncture as a potential therapeutic strategy effective in restoring microbial homeostasis, modulating neurotransmitters like serotonin and GABA, and reducing neuroinflammation. While clinical evidence supports acupuncture's benefits for sleep quality and relapse reduction, more detailed studies are needed to standardize treatments and validate integrative approaches for insomnia.

### Anorexia nervosa/bulimia nervosa

Yu et al.'s bidirectional MR analysis established causal relationships between 18 gut microbial taxa and anorexia nervosa (AN)/bulimia nervosa (BN). Family *Lachnospiraceae* exhibits opposing effects: specific genera increase AN risk yet confer protection against BN. These findings underscore microbiota's complex role in eating disorders and offer novel targets for probiotic therapies, notwithstanding limitations in microbial genome-wide association studies that require further validation.

## Neurodevelopmental disorders

### Autism spectrum disorder

Li et al. conducted a study employing 16S rRNA sequencing to examine 957 children with autism spectrum disorder (ASD) aged 2–12 years, alongside 161 HC within the same age range in China. This extensive research elucidates gut dysbiosis among Chinese children with ASD, demonstrating: reduced α-diversity, altered microbial abundances (*Faecalibacterium* enrichment and *Prevotella_9* depletion), and disrupted metabolic pathways, including folate synthesis. These findings substantiate the involvement of the gut-brain axis in ASD pathogenesis and underscore the potential of microbiota as biomarkers, necessitating further longitudinal studies for validation. Using a 16p11.2 microduplication mouse model of autism, Fu Z. et al. identified gut dysbiosis and disrupted microbial neurotransmitter networks. Key findings comprise the following: reduced microbial biodiversity, depletion of *Faecalibaculum*, elevated histaminergic metabolites, and correlations between specific bacterial shifts and autism-like phenotypes. The research highlights the gut-brain axis's role in neurodevelopmental disorders and proposes targeting histamine metabolism as a potential therapy for 16p11.2 microduplication- associated ASD. Ying et al.'s bibliometric analysis delineates the evolving ASD-gut microbiota research landscape (2000–2021). Analysis of 100 foundational publications reveals: surging annual citations peaking in 2021, leading contributions from the United States and Ireland, and dominant themes of short-chain fatty acids and MGBA mechanisms. Although 62% of the publications are reviews, pivotal experimental studies, notably Hsiao's 2013 *Cell* paper, demonstrate therapeutic promise. The analysis also maps collaborative networks and emerging research areas, despite being limited to one database, offering a roadmap for future research.

## Other brain diseases or related diseases

Pasam and Dandekar studied the impact of controlled cortical impact (CCI) injury on gut microbiota in male and female mice. Post-CCI dysbiosis was characterized by significant reductions in *Lactobacillus helveticus* and *L. hamsteri* in female. Cross-sex FMT indicated that female recipients of male microbiota exhibited enriched neuroprotective *Lactobacillus*, whereas male recipients shoed increased abundance of *Alistipes* and *Ruminococcus*. These results highlight sex-specific microbial responses to traumatic brain injury and suggest *Lactobacillus*-targeted interventions as a mechanisnm-driven strategy for sex-specific neurorecovery.

Using a Mendelian randomization study, Zeng C. et al. established causal effects of specific gut microbiota on trigeminal neuralgia (TN). The analysis identified *Butyricimonas, Bacteroidales* S24.7 group, and an unclassified genus as significant risk of TN, contradicting the presumed exclusively beneficial role of *Butyricimonas*. Sensitivity analyses supported these results, and reverse MR analysis excluded reverse causation. Despite limited generalizability from Finnish GWAS data, this study suggests a gut-brain connection in TN's pathogenesis, emphasizing the need for randomized trials to explore mechanisms and therapeutic options.

Zhang J. et al.'s review highlights the crucial role of peripheral dysfunction, including gut microbiota imbalance, liver metabolite changes, and cholinergic pathway issues, in sepsis-associated encephalopathy (SAE). The work elucidates novel mechanisms by which neuroinflammation, blood-brain barrier disruption, and inter-organ crosstalk exacerbate SAE progression. The study emphasizes multiorgan interactions as therapeutic targets, suggesting mechanism-based interventions including fecal microbiota transplantation, short-chain fatty acids, and vagus nerve modulation. This comprehensive view moves SAE management beyond traditional central nervous system-focused approaches.

While primarily a digestive disorder, functional dyspepsia (FD) exhibits neuropsychiatric linkages. Zhang X. et al.'s systematic review highlights duodenal microbiota dysbiosis as a key factor in FD pathogenesis and gut-brain axis dysfunction. Analysis of 391 FD patients revealed symptom-associated alterations: increased *Streptococcus* abundance coupled with decreased *Prevotella* and *Selenomonas*. *Staphylococcus*-driven inflammation may exacerbate FD progression. Paradoxically, despite stable α-diversity, microbial imbalances correlated with symptom severity and quality of life. The authors advocate for expanded studies to validate the therapeutic targets and elucidate underlying mechanisms.

## Final remarks

This Research Topic highlights the causal involvement and therapeutic potential of gut microbiota in brain disorders through animal models. Mendelian randomization studies identified disease-specific microbial signatures, notably *Desulfovibrionaceae* elevating Alzheimer's disease risk and *Butyricimonas* attenuation Parkinson's dementia risk. Interventions including fecal microbiota transplantation and electroacupuncture improved cognitive/motor deficits by restoring microbial homeostasis, enhancing gut-brain barrier integrity, and suppressing neuroinflammation. The MGBA emerges as a central pathway in epilepsy, depression, and autism spectrum disorder. Current limitations encompass population homogeneity, therapeutic standardization deficits, and translational gaps. Future priorities include multi-omics integration to elucidated metabolite-neural circuits, longitudinal biomarker validation, and randomized trials for personalized microbial therapeutics.

## References

[B1] Cryan J. F. Dinan T. G. (2012). Mind-altering microorganisms: the impact of the gut microbiota on brain and behaviour. Nat. Rev. Neurosci. 13, 701–712. 10.1038/nrn3346 22968153

[B2] Gao X. Wang Y. Meng H. Li S. Jiang H. Zhang Z.. (2023). Acupuncture for brain diseases: conception, application, and exploration. Anat. Rec. 306, 2958–2973. 10.1002/ar.24884 35195374

[B3] Neufeld K. M. Kang N. Bienenstock J. Foster J. A. (2011). Reduced anxiety-like behavior and central neurochemical change in germ-free mice. Neurogastroenterol. Motil. 23, 255–264.e119. 10.1111/j.1365-2982.2010.01620.x 21054680

